# Compositional stability of peat in ecosystem-scale warming mesocosms

**DOI:** 10.1371/journal.pone.0263994

**Published:** 2022-03-02

**Authors:** Mackenzie R. Baysinger, Rachel M. Wilson, Paul J. Hanson, Joel E. Kostka, Jeffrey P. Chanton

**Affiliations:** 1 Department of Earth, Ocean and Atmospheric Sciences, Florida State University, Tallahassee, Florida, United States of America; 2 Environmental Sciences Division, Climate Change Science Institute, Oak Ridge National Laboratory, Oak Ridge, Tennessee, United States of America; 3 School of Biological Sciences, Georgia Institute of Technology, Atlanta, Georgia, United States of America; 4 School of Earth and Atmospheric Sciences, Georgia Institute of Technology, Atlanta, Georgia, United States of America; Tennessee State University, UNITED STATES

## Abstract

Peatlands historically have acted as a C sink because C-fixation rates exceeded the rate of heterotrophic decomposition. Under future warmer conditions predicted for higher latitudes, however, that balance may shift towards higher rates of heterotrophic respiration leading to the release of previously stored C as CO_2_ and CH_4_. The Spruce and Peatlands Response Under Changing Environments (SPRUCE) experiment is designed to test the response of peatlands to climate forcing using a series of warmed enclosures in combination with peat below-ground heating from 0 to +9°C above ambient conditions. This experimental design allowed a test of chemical changes occurring within peatland soils following five years of warming. We analyzed samples in the uppermost 2m of peat using Fourier Transform Infrared Spectroscopy (FT-IR) to quantify the relative abundance of carbohydrate and aromatic compounds in the peat. The peat soils were subjected to deep peat heating (DPH) beginning in June of 2014 followed by whole ecosystem warming (WEW) in August of 2015. We found that the relative amounts of labile and recalcitrant chemical compound groups across the full peat depth interval did not significantly change after five years of exposure to warming. This appears the case even though previous studies have shown that net C losses and loss of bulk peat mass to be instability over that time period. Results suggest that the current store of carbon in peatlands are largely compositionally stable leading to no changes the in the ratio of chemical moieties on the initial four-year timescale of this experiment.

## 1 Introduction

Peatlands are large terrestrial carbon banks that represent 30% of the world’s soil carbon [1]. They are of particular interest because they contain a quantity of carbon roughly equal to the quantity present in the preindustrial atmosphere [2]. High latitude peatlands have been accumulating C since deglaciation and have been important in the global carbon cycle throughout the Holocene [3, 4]. Over this period, carbon fixation and net accumulation into high latitude peatlands has outpaced decomposition due to cold temperatures, high water tables, and low O_2_ availability. More than 90% of peatland carbon stocks are found in high northern latitudes [2] where they are expected to experience the effects of climate change to a greater degree than peatlands closer to the equator [5]. The ability of peatlands to remain as carbon sinks in a warming climate is uncertain. With warmer climate, peatland carbon stocks could be released as CO_2_ and CH_4_ which would lead to greater atmospheric forcing, warmer temperatures and even greater subsequent soil carbon release [6–8].

Respiration and fermentation of organic material in the subsurface of peatlands is mostly anaerobic and results in the production of the end products CO_2_ and CH_4_. The decomposition rate and the ratio of the produced gases is influenced by many factors, but is largely impacted by the terminal electron accepter (TEA) which supports microbial metabolism. High energy yield TEAs increase the amount of CO_2_ produced, while under TEA-depleted, anaerobic conditions, CO_2_ itself can act as a TEA to produce CH_4_. However, even under completely methanogenic conditions decomposition rates and CO_2_/CH_4_ ratio can vary due to the oxidation state of the organic matter itself [9–13]. There is increasing evidence that peatlands become increasingly methanogenic under warmer conditions [14, 15]. We hypothesize that this may be due to changes in the peat composition during anaerobic metabolism that increasingly favors methane production. For example, high oxidation state organic C is thought to be highly bioavailable [12, 13] and may be used up first leaving behind an increasing peat C pool of lower oxidation state organic matter that favors CH_4_ over CO_2_ production. This effect could exacerbate climate warming since CH_4_ has a much stronger global warming potential than CO_2_ [16, 17], thus creating a positive feedback between peatlands and climate.

Because microorganism enzyme action breaks down the components of peat at different rates the relative abundance of carbohydrates and aromatic compounds is a useful metric for determining levels of humification/decomposition [7, 18–21]. Carbohydrates (O-alkyl-C) are formed in plants as cellulose and are more bioavailable than other compound classes such as lignin/aromatics [13, 22]. The Nominal Oxidation State of Carbon (NOSC, [12]) allows an estimate of the energetic potential and bioavailabity of organic matter. Degradation of high NOSC organic matter (i.e., carbohydrates) results in a greater energy yield relative to degradation of compounds with lower NOSC. Under anaerobic conditions, higher NOSC compounds appear to be used preferentially leaving behind a pool of increasingly less bioavailable, more reduced compounds (e.g., lipids and unsaturated hydrocarbons) [22, 23]. This phenomenon can be readily observed with increasing depth in the peat column; more mature peat at depth contains a lower percentage of biodegradable organic matter (carbohydrates) than the recently formed peat closer to the surface [10, 19, 20, 22, 24]. A similar trend has also been found for surface peats along a gradient of latitude; carbohydrate content declines under the warmer conditions found closer to the equator [24] because of higher decomposition rates at warmer temperatures.

Hodgkins et al., [24] postulated that the greater amount of carbohydrate in high latitude peats renders the carbon stored there vulnerable to warming. With warming such soils could undergo losses of the carbohydrate-rich fraction. To test this hypothesis, we quantified the carbohydrate fraction of peat soils under varying degrees of in situ warming, from 0 to 9°C above ambient, over five years. In this study, we sampled peat cores from an artificially heated bog (Spruce and Peatland Responses Under Changing Environments (SPRUCE) located in the Marcell Experimental Forest, MN) and measured their carbohydrate and aromatic content using Fourier Transform Infrared Spectroscopy (FTIR) analyses following the quantification method of Hodgkins et al. [24]. We hypothesized that the carbohydrate component groups would be lost from the soil organic matter at a greater rate with increasing temperature relative to other peat soil components as they are the most labile part of the sample, and would therefore be consumed preferentially by the microbial community in the soil matrix.

## 2 Methods

### 2.1 Study sites and sample collection

Ethics Statement: SPRUCE is collaboration between the USDA Forest Service and the Department of Energy through Oak Ridge National Laboratory (ORNL). The interagency agreement between the two agencies (2011-IA-123) allows sample collection for ORNL scientists and other SPRUCE collaborators. To evaluate the impacts of temperature treatment on peat carbohydrate degradation, samples were analyzed from a suite of 2 m-long peat cores collected from an ombrotrophic peatland in Minnesota (USA), where the Spruce and Peatland Responses Under Changing Environments (SPRUCE) project is being conducted (for schematic and site description see [6, 25, 26]. SPRUCE is an ecosystem-scale study that warms the vegetation and peatland soil profile to a depth of 2 m within 12.8-m diameter open-top enclosures. The peat was heated first at– 2 m for a period of 1 year starting in summer 2014 (deep peat heating phase), then heating of the overlying air began in August 2015 starting the whole-ecosystem warming phase of the experiment. Five temperature differentials have been obtained (ambient, +2.2, +4.5, +6.8, and +9°C). There were duplicate enclosures subjected to the same temperature at both ambient and ~ + 500 p.p.m.v. above ambient atmospheric CO_2_ concentrations [26]. Each of these enclosures were sampled for this project. One core was taken from each enclosure and divided into 11 partitions representing 10 cm depth increments up to 50cm, where they were then partitioned every 25cm to 200cm below surface. Whole-ecosystem above ground warming (WEW) was initiated August 2015, following 14 months of deep-peat heating that began June 2014. Water table was measurements using automated sensors have been taken daily beginning in 2015. Soil cores within each enclosure were collected first in August of 2014 and in August 2019, after 4 years of whole ecosystem warming and 5 years of peat-heating.

### 2.2 Sample analysis

**Fig 1:**
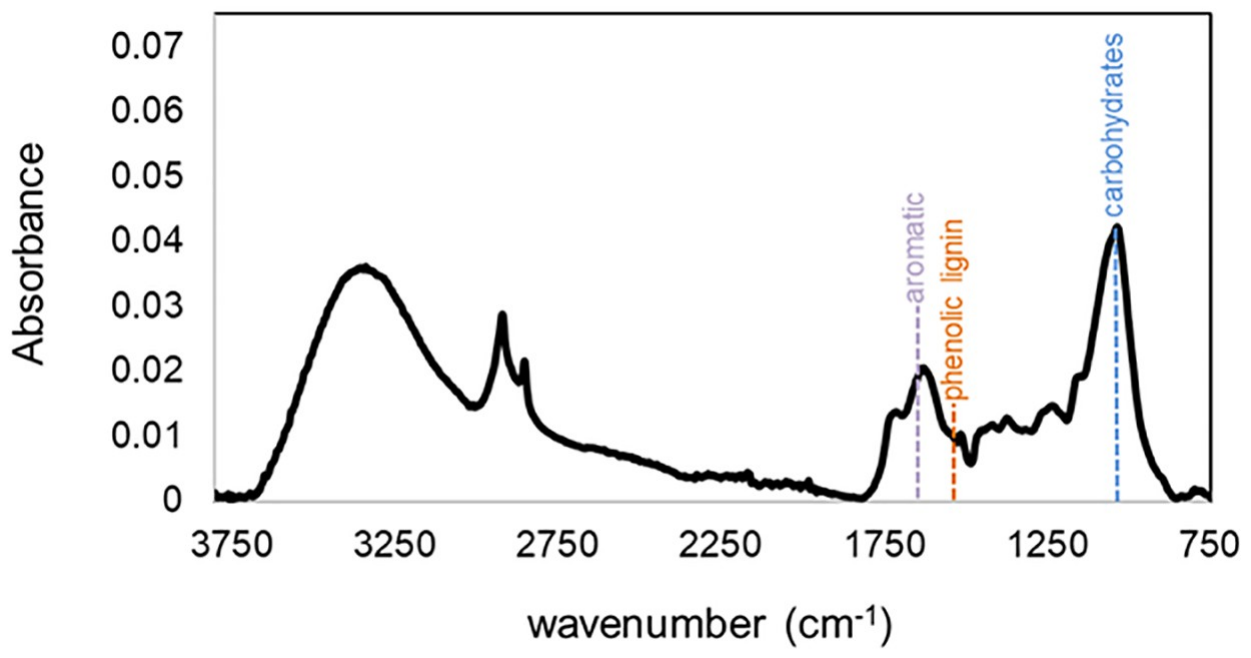
FTIR spectra of peat collected from an ambient enclosure at SPRUCE in September 2014 (depth = 25 cm). Following Hodgkins et al., [24], we focused on the peaks at 1040 cm^-1^ (O-alkyl of carbohydrates), 1510 cm^-1^ (phenolic lignin- like structures), and 1630 cm^-1^ (aromatic structures because these change in systematic ways in peat soils with depth at a single location and with latitude in surface soils. By normalizing the peaks to the area below the spectral curve, we can quantitatively observe changes in the chemistry of our samples.

After transport from the field, peat subsamples, including roots, were freeze-dried to ensure shelf stability. The peat was then ground to a homogenous powder in a Spex Sampleprep 5100 mixer-mill. To evaluate the response of peat organic carbon fractions to the ecosystem-scale heating, Fourier Transform Infrared Spectroscopy (FTIR) analyses were conducted on each depth increment (0-10cm, 10-20cm, 20-30cm, etc) of the peat cores collected from all of the 10 treatment enclosures in both 2014 and 2019, resulting in 195 individual samples. FTIR analysis was chosen because it allows us to semi-quantitatively measure the functional composition of the sample [24, 27] (Fig 1). FTIR spectra were collected with a PerkinElmer Spectrum 100 FTIR spectrometer fitted with a CsI beam splitter and a deuterated triglycine sulfate detector. Transmission-like spectra were obtained with a Universal ATR accessory with a single-reflectance system and made from a zinc selenide (ZnSe)/diamond composite. Samples were placed directly on the ATR crystal, and force was applied so that the sample came into good contact with the crystal. Spectra were acquired in % transmittance mode between 4000–650 cm ^−1^ (wavenumber) at a resolution of 4 cm ^−1^, and four scans were averaged for each spectrum. The spectra were ATR-corrected to account for differences in depth of beam penetration at different wavelengths, and then baseline-corrected, with the instrument software. Spectra were then converted to absorbance mode for subsequent data analysis. In this study, we followed the FTIR data processing method of Hodgkins et al [24] (available here: https://github.com/shodgkins/FTIRbaselines) that allows for more thorough quantification of compound classes. Instead of normalizing peak heights relative to other peaks via humification indices, we isolated each compound by normalizing peaks to the integrated area of the entire spectrum. Normalized peak heights are then reported and used for analysis.

Employing the calibration method from Hodgkins et al. [24], the normalized peak heights can then be used to quantify the presence of molecular groups in a given sample. Knowing the percent composition of carbohydrates, etc. in allows us to see how that composition changes over time of warming exposure. Post-treatment peat samples (2019) were compared to pre-treatment samples (2014) to measure shifts (if any) in molecular group presence.

## 3 Results

**Fig 2:**
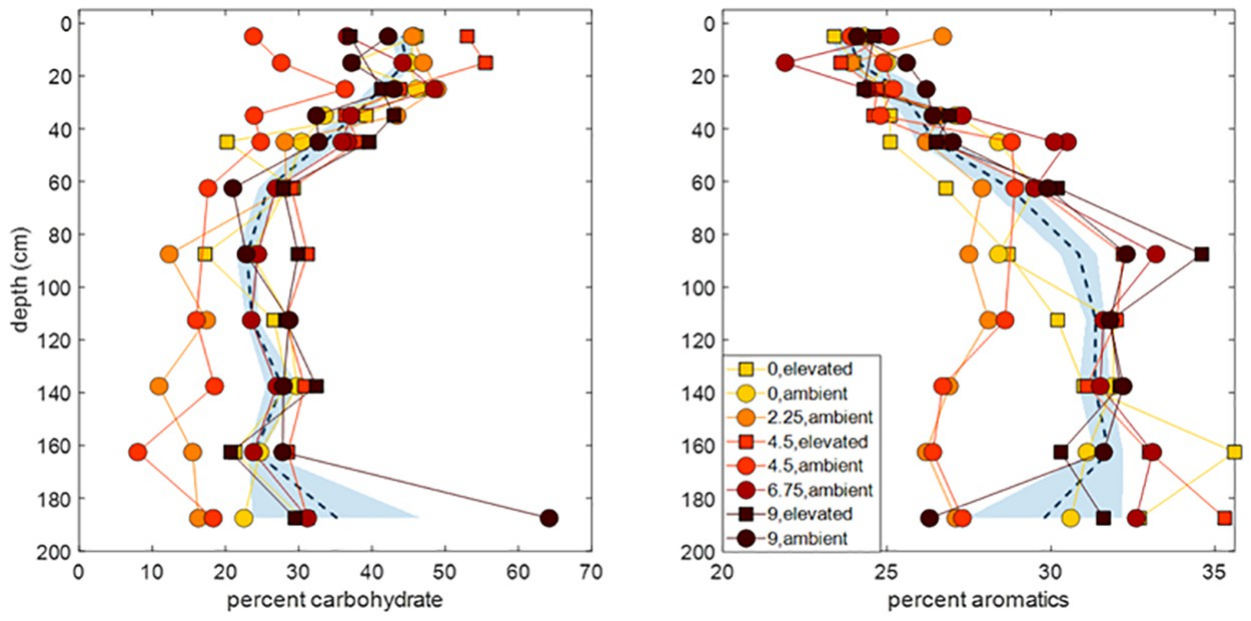
Organic matter composition. Percent carbohydrate and aromatic content in samples compared from each temperature treatment in 2019 compared to the 2014 pre-heating values, circles indicate ambient CO_2_ and squares indicate elevated CO_2_ treatments. Dashed lines indicate the locally weighted polynomial regression (LOESS) of the pre-heating (2014) values across all enclosures with the ±95% confidence interval indicated by the blue shaded area around each line (MATLAB 2020a). Results for 2019 are plotted as individual symbols for each temperature treatment as indicated in the legend. decreased with depth, with no discernable difference between pre-heating and five-years into the warming. Panel A. Carbohydrate content decreased from surface to -70cm, where carbohydrate content stabilized or slightly increased. Panel B. Aromatic content in samples increased with depth, with no discernable difference between pre-heating and five-years into the warming.

Carbohydrate content in the peat column generally decreased with depth down to -70 cm below which the % carbohydrate remained stable or even increased slightly (Fig 2A). The composition ranged from 23–55% at the surface and showed a negative relationship to depth from surface. The samples taken from -200 cm contained 11–31% carbohydrate content. In contrast, the aromatic content increased with depth, to about -70 cm below which the %aromatics remained relatively stable down to 2 m (Fig 2B). The aromatic content ranged from 23–26% at the surface, and steadily increased in content to 26–36% at the -200cm depth (Fig 2B). We compared the carbohydrate and aromatic content of the 2014 samples to the 2019 samples and found that the chemistry of the peat samples did not significantly change after 5 years of exposure to the ecosystem-scale warming. (carbohydrate content p-value = 0.73; aromatic content p-value = 0.84 by Wilcox-test; Fig 2A and 2B).

**Fig 3:**
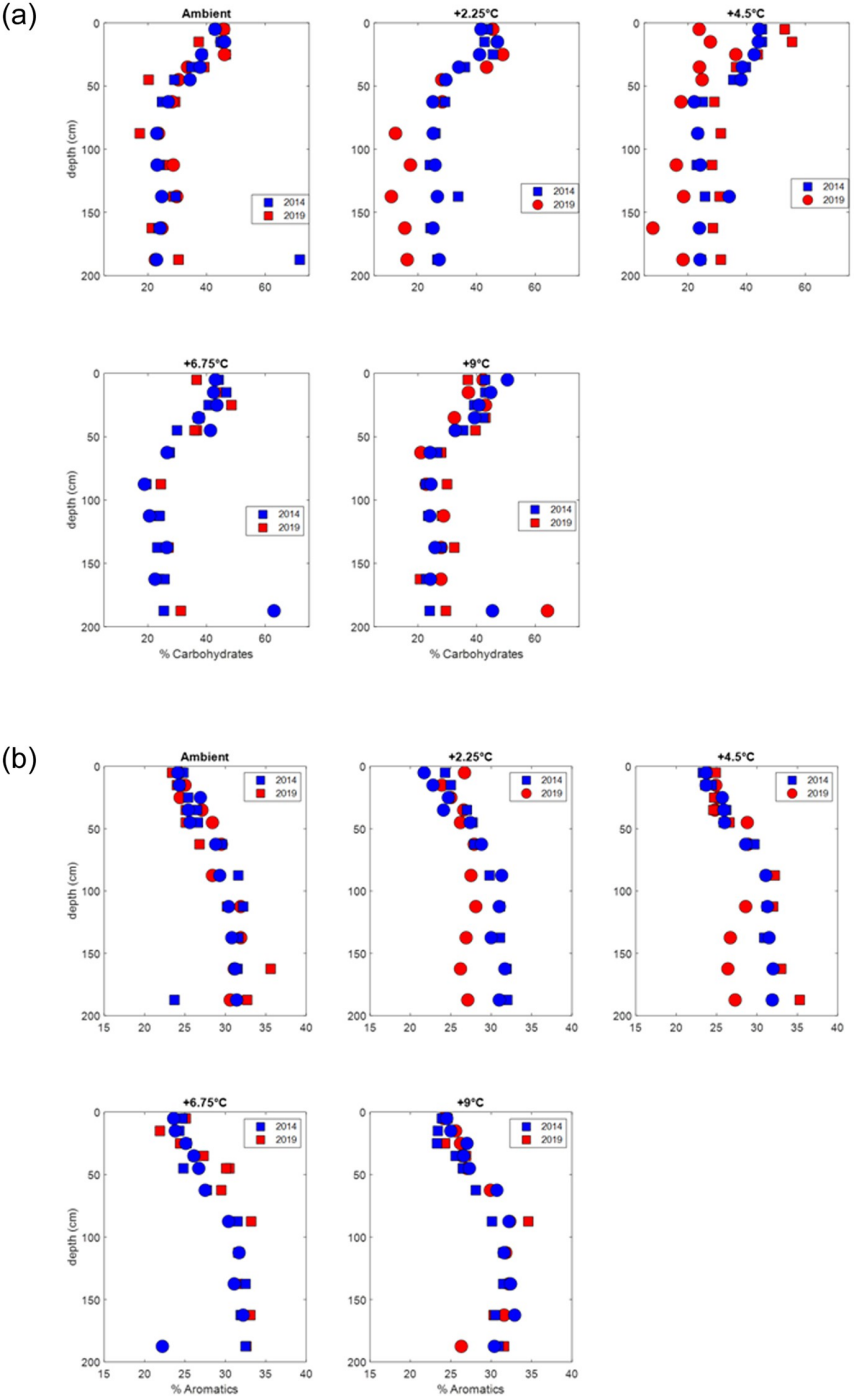
Carbohydrate and aromatic content versus depth. Carbohydrate content in peat cores taken from each temperature treatment in duplicate heated enclosures (two cores per temperature treatment) from the beginning of deep peat heating (2014) and after 5 years of warming (2019) (upper panel). Lower panel, aromatic content. Circles are the ambient CO_2_ and squares are the elevated CO_2_ treatments.

**Fig 4:**
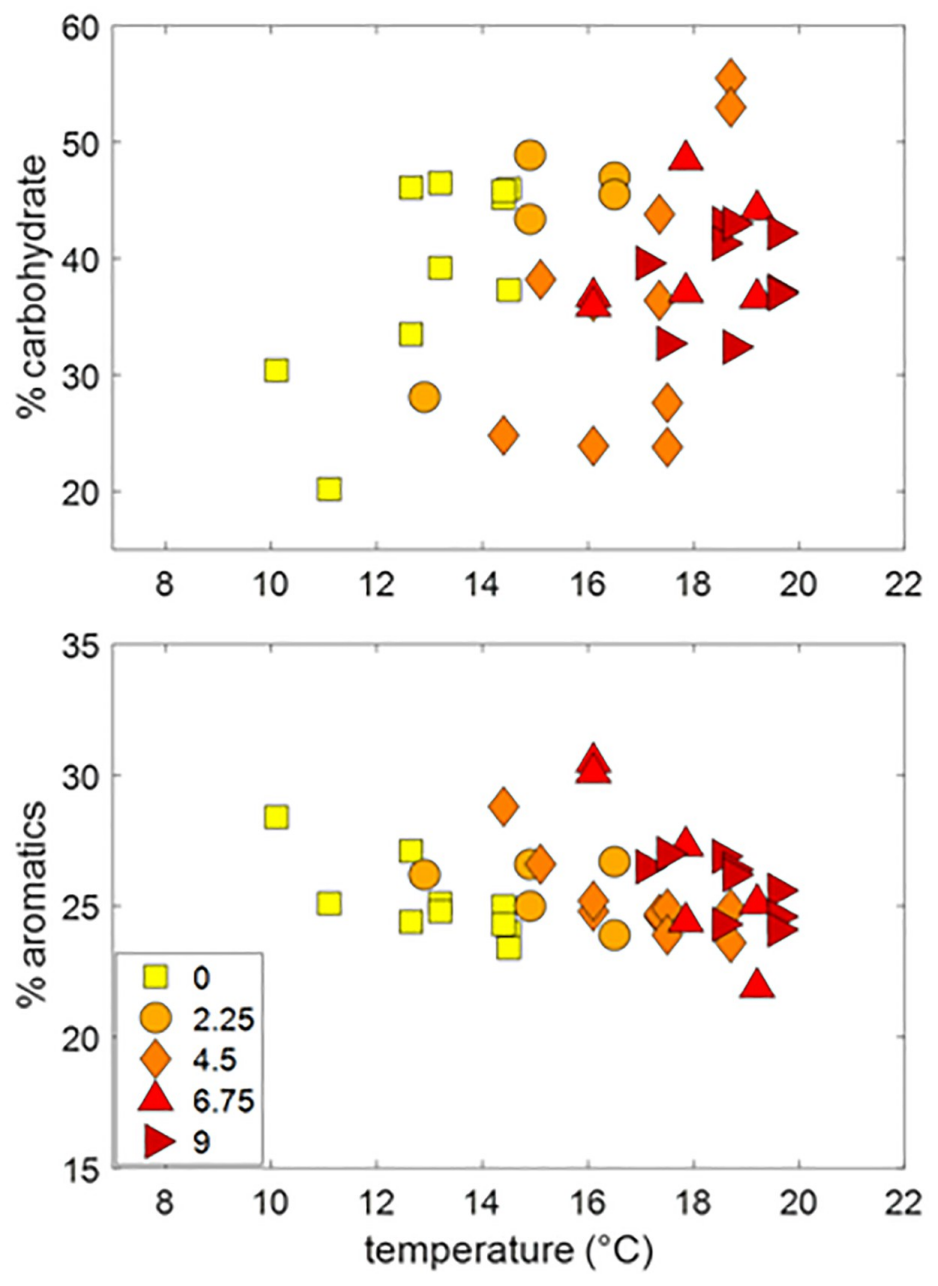
Regressions versus temperature. Temperature regressions of percent carbohydrate (panel A) and percent aromatics (Panel B) in the surface peat (≤ 50 cm) collected in 2019. Data are plotted against the actual averaged measured temperature at each peat depth averaged for the month of August 2019. Symbol colors indicate the temperature treatment differential. Regression analyses were not significant for either % carbohydrate or % aromatics (p > 0.1).

To look at temperature-specific changes in the composition of the samples we plot the carbohydrate and aromatic content data by depth from surface for the individual enclosure treatments (Fig 3). Samples were taken in duplicate—two individual domes were subjected to identical heat treatment, as represented by the color in Fig 3. The Spruce experiment is a regression-based design (Fig 4) and includes a broad range of temperature levels to produce data to characterization response curves for application to ecosystem and Earth system models [26].

In the ambient and +2.25°C treatments the 2019 measurements closely matched those from the beginning of the warming treatment in 2014. In the +4.5, 6.75 and +9°C treatments the surface %carbohydrates were frequently, but not consistently, lower in 2019 relative to the starting 2014 values (Fig 4). These differences generally diminished deeper in the peat profile. Sample depth was a larger indicator of peat carbohydrate content relative to the heat treatment group for the 5 years of in situ heating.

Additionally, we plot the aromatic content by depth of sample (Fig 3). We did not see variance in the amount of carbohydrate content or aromatic content in the surface peat with differing temperature treatments (Fig 4). These findings are consistent throughout the entirety of the peat column sampled.

**Fig 5:**
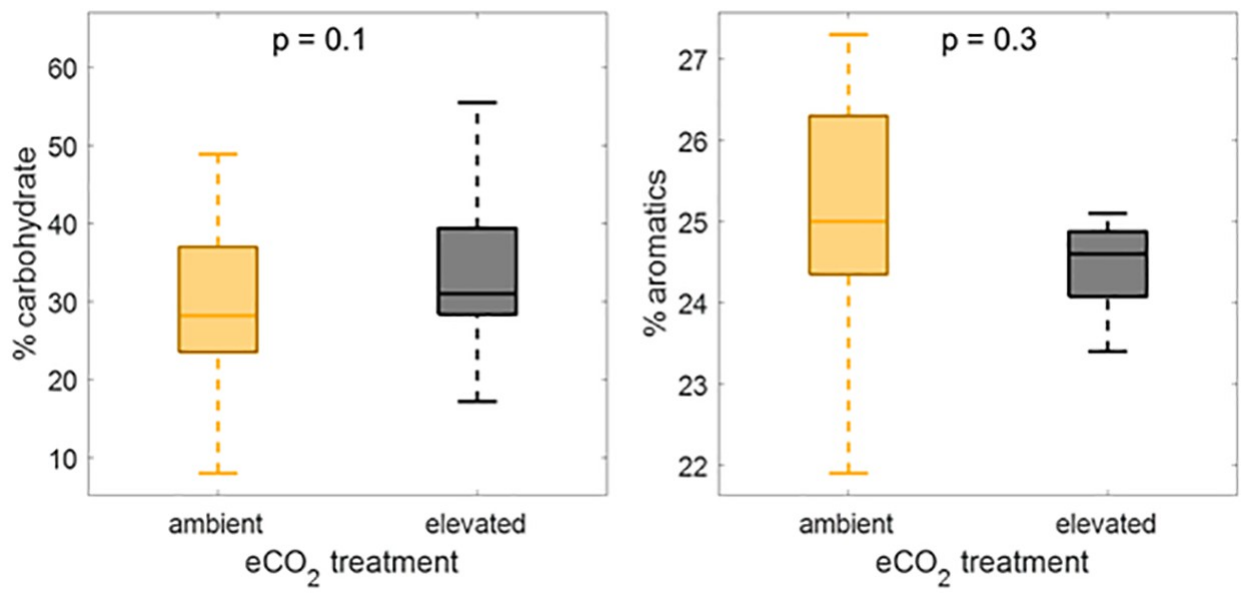
Effect of CO_2_ treatment. Comparison of percent carbohydrate (panel A) and percent aromatics (panel B) in the ambient and elevated CO_2_ treatments. Values for the boxplots were taken only from 2019, after the eCO_2_ treatment had begun and only from surface peat (<50 cm) where the majority of root inputs occurs. Results of T-tests comparing elevated and ambient values are given in the top of each panel.

We compared the percent carbohydrate and percent aromatics in the elevated and ambient plots for 2019 (Fig 5). Although the median percent carbohydrate was slightly higher in the elevated CO_2_ treatment relative to the controls, the differences were not significant (T-test, p = 0.1). Similarly, % aromatics were not significantly different between the ambient and the elevated CO_2_ plots.

## 4 Discussion

In this study we sampled peat cores from the SPRUCE experimental treatments in northern Minnesota. We hypothesized that over that time period we would observe a decrease in the carbohydrate content of surface peats as carbohydrates preferentially fueled microbial respiration and as surface peat decomposed at a higher rate at higher temperatures [6]. In addition, we also measured changes in the aromatic content which represents the aromatic rings of quinine and amide compounds in the samples. Aromatic groups are more difficult to decompose compared with the more labile carbohydrates, thus they are expected to increase in the peat during decomposition as more labile components are preferentially removed.

The lack of chemical response of surface peat to temperature increase was surprising as previous work has clearly shown that the system is becoming increasingly methanogenic with warming [14, 28, 29]. Additionally, while early work at SPRUCE failed to find evidence of peat contribution to GHG production [14], more recent studies do show that the peat itself is being degraded as the experiment progresses [6, 29] especially at the higher temperature enclosures, and that an increasing fraction of decomposition supports methane production. We hypothesized that this increasingly methanogenic GHG production was the result of peat decomposition that preferentially removed higher NOSC compounds first leaving behind an increasingly less oxidized carbon pool for decomposition via methanogenesis. The lower NOSC residual would then result in more methane production relative to CO_2_, as dictated by electron balance. However, results in this paper do not yield evidence that this is the case. There does not appear to be an observable preferential loss of high NOSC (e.g., carbohydrate) compounds with temperature treatment (Fig 4) at least over a five-year period during which time methane production rates have increased relative to CO_2_ production rates. Therefore, the reason for this shift must be due to kinetic factors affecting rates of degradation processes differentially.

The elevated CO_2_ treatment stimulates primary production which increases root production and exudation especially in the rooting zone (< 30 cm) [30]. We expected that this increase in production would result in increased carbohydrate content in the shallow peat within the elevated CO_2_ treatments. We observed that the percent carbohydrate in the elevated CO_2_ treatment was slightly, though not significantly higher than in the ambient CO_2_ plots (Fig 5). One explanation for this result is that the large C pool in peat is shifting, but too slowly for us to observe on the timescale of this experiment. Further monitoring is necessary to determine whether this trend, of increasing percent carbohydrate in the surface peat, continues or becomes stronger with time. Increasing inputs of carbohydrates in the elevated CO_2_ treatments may confound our interpretation of changing carbohydrate content with temperature if new carbohydrates are being input to the system as decomposition is also stimulated to remove those carbohydrates. There were fewer carbohydrates in the +2.25°C and +4.5°C treatments, but that trend was not consistent at the higher temperature treatments (Fig 3). Recent lab-based studies show that decomposition rate at these temperatures does increase systematically across all of these temperatures (Wilson et al., 2016; Hopple et al., 2020), but a microbial preference in peat composition simply cannot be observed on the timescale of this 5-year study.

Nevertheless, the depth profiles from the peat (Figs 2 and 3) provide evidence that, in principle, as decomposition progresses, carbohydrates are preferentially removed and aromatic content increases. However, our results show that this process of microbial organisms preferentially removing carbohydrates takes more time than the scope of this experiment. Such observations are consistent with other peats where these measurements have been conducted (e.g., [20, 24]). On the longer time scales captured by our depth profiles carbohydrates are removed and aromatics increase, but the peat C pool is so large that over the timescale of our experiment we might not have the resolution to capture changes in the peat composition with temperature. Based on radiocarbon profiles at the SPRUCE site, these changes occur on a timescale of 100’s to 1000 years [31]. These rates are consistent with those shown by Hodgkins et al. [24] as well.

The stability of the peatland’s carbohydrate composition below 70 cm is not surprising, as both acrotelm and catolem peat has been shown to be stable in its waterlogged, anoxic state [14, 32]. Water table depths during the SPRUCE experiment from 2015 through 2020 have been driven by a balance between precipitation inputs and evapotranspiration losses (as expected). While those losses are higher under the warmest treatments, an abundance of ambient precipitation prior to the 2021 growing season largely sustained water tables high within the acrotelm layer of the S1-bog peatland. The net impact of this extended “wet” period is that most of the peat profile has supported anoxic conditions. Full water table data are available at [33]. That data set includes data through 2019 but will be appended with future observations for 2020, 2021 and all future years of the SPRUCE experiment. The stability of the S1-bog’s water table height throughout the entirety of the WEW measurements, has contributed to keeping the organic matter at depth in long-term storage.

Peat C decomposition is mediated by microbial enzyme activity, which in turn is strongly influenced by environmental factors such as temperature, substrate quality, and moisture [34]. Changing enzyme activities, such as the decline in β-D-glucosidase activity with depth observed by Hill et al. [35] suggest that peat C becomes increasingly less reactive with age. The enzyme β-D-glucosidase is the primary observed C-acquiring enzyme across a range of wetland types and interestingly, the abundance of this enzyme suggests that despite the large pool of C in peat, peatland microorganisms appear to be C-limited (rather than N or P limited; Hill et al., [36]) consistent with large quantities of the C being sequestered in less bioavailable forms. Nevertheless, the breakdown of compounds at depth does occur, but at a slower rate than in the upper surface layer [32]. For example, polyphenol oxidases, enzymes which break down lignin and other phenolic compounds, increase with depth in the peat profile [35], although it should be noted that the opposite trend was observed by Steinweg et al., [34]. In the peat column, depth has been shown to drive the microbial community [14, 37, 38] and resulting enzyme profiles [34]. Temperatures in the deep peat are more stable relative to the surface resulting in deep microbial enzymes that appear to be less responsive to warming temperatures suggesting that, while surface enzyme activity does appear sensitive to warmer temperatures [39], there is little change in C decomposition with temperature in the catotelm [14, 34]. Our results in Fig 2 are consistent with faster removal of carbohydrate C in the surface peat, followed by relatively stable conditions in the catotelm below 40 cm.

In conclusion, our results indicated that, while net peat C loss does occur [6], the current store of carbon in the SPRUCE peatland is compositionally stable, at least on the timescale of this experiment, as the samples did not show a significant change in absorbances at the key wavelengths from 2014 to 2019. Labile components, primarily the carbohydrate groups at the 1040 cm^-1^ peak, did not respond to the increased warming over the five years of applied belowground temperature treatment. Aromatic groups (1515 and 1630 cm^-1^) also did not show a response to the five years of applied treatment covered in this study.
